# Supplementary figures and images for: ResMem-Net: memory based deep CNN for image memorability estimation
Source: PeerJ Comput Sci. 2021 Nov 5;7:e767. doi: 10.7717/peerj-cs.767 (PMC8594589; doi:10.7717/peerj-cs.767)

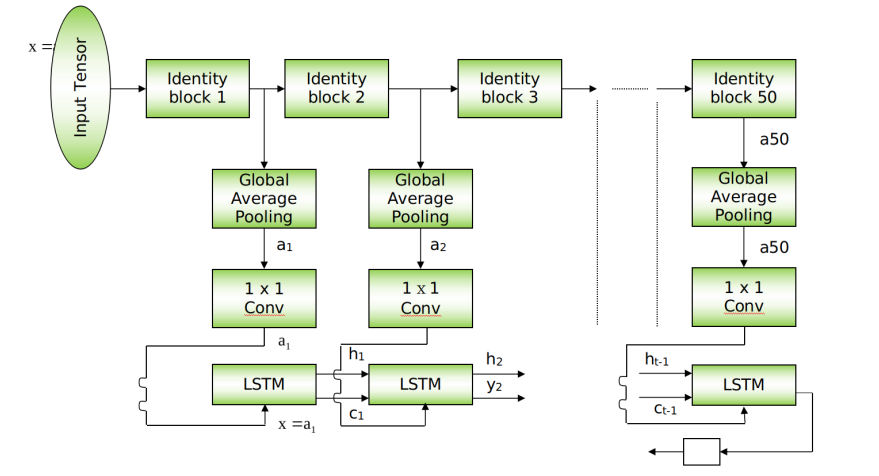

Supplement: Supplemental Information 1 [file peerj-cs-07-767-s001.zip › ResMemNet-master/Photos/model.png]
